# Comparing the Effect of Neostigmine and Metoclopramide on Gastric Residual Volume of Mechanically Ventilated Patients in Intensive Care Unit: A Double-Blind Randomized Clinical Trial

**DOI:** 10.1155/2021/5550653

**Published:** 2021-08-16

**Authors:** Alireza Rahat-Dahmardeh, Sara Saneie-Moghadam, Masoum Khosh-Fetrat

**Affiliations:** ^1^Department of Anesthesiology and Critical Care, School of Medicine, Zahedan University of Medical Sciences, Zahedan, Iran; ^2^Department of Anesthesiology and Critical Care, Khatamolanbia Hospital, Zahedan University of Medical Sciences, Zahedan, Iran

## Abstract

**Introduction:**

The gastric residual volume (GRV) monitoring in patients with mechanical ventilation (MV) is a common and important challenge. The purpose of this study was to compare the effect of neostigmine and metoclopramide on GRV among MV patients in the intensive care unit (ICU).

**Methods:**

In a double-blind randomized clinical trial, a total of 200 mechanically ventilated ICU patients with GRV > 120 ml (6 hours after the last gavage) were randomly assigned into two groups (A and B) with 100 patients in each group. Patients in groups A and B received intravenous infusion of neostigmine at a dose of 2.5 mg/100 ml normal saline and metoclopramide at a dose of 10 mg/100 ml normal saline, within 30 minutes, respectively. GRV was evaluated 5 times for each patient, once before the intervention and 4 times (at 3, 6, 9, and 12 hours) after the intervention. In addition, demographic characteristics including age and gender, as well as severity illness based on the sequential organ failure assessment score (SOFA), were initially recorded for all patients.

**Results:**

After adjusting of demographic and clinical characteristics (age, gender, and SOFA score), the generalized estimating equation (GEE) model revealed that neostigmine treatment increased odds of GRV improvement compared to the metoclopramide group (OR = 2.45, 95% CI: 1.60-3.76, *P* < 0.001). However, there is a statistically significant time trend (within-subject differences or time effect) regardless of treatment groups (*P* < 0.001).

**Conclusion:**

According to the results, although neostigmine treatment significantly improved GRV in more patients in less time, within 12 hours of treatment, all patients in both groups had complete recovery. Considering that there was no significant difference between the two groups in terms of side effects, it seems that both drugs are effective in improving the GRV of ICU patients.

## 1. Introduction

Early enteral nutrition (EN) is the standard metabolic support in critically ill patients under mechanical ventilation (MV). In patients whose nutritional requirements cannot be met by oral feeding, enteral feeding is the preferred route of nutrition support [1, 2]. One big problem in mechanically ventilated ICU patients is delayed gastric emptying [3–5]. Evidence showed that more than 50% of patients in ICU have gastric dysmotility, which leads to slow gastric emptying and high gastric residual volume (GRV) and is associated with increased mortality in these patients [6–9]. Delayed gastric emptying can induce several problems, which can influence ICU outcomes and lead to inadequate caloric intake or infrequent usage of EN. In addition, nausea, regurgitation, and aspiration can increase the risk of ventilator-associated pneumonia (VAP) and consequently increase the length of hospital stay [10–14]. Thus, monitoring of GRV is recommended to decrease the incidence of these complications. In cases of high GRV, decreasing the volume of enteral feeding or the formula osmolality seems to be necessary.

Till now, there have been surgical procedures and pharmacological methods to facilitate this process and decrease the GRV in patients, but each of them has their limitations [15, 16]. different kinds of drugs including metoclopramide, erythromycin, and cisapride are used, but none of them had conclusive evidence of better effects on each other [17]. One of the drugs that have been used in this field recently is neostigmine [18]. Studies have reported different results about the effectiveness of neostigmine on the tolerance of enteral feeding, especially in patients in the ICU [19, 20]. While the efficacy of neostigmine on the postoperative ileus has been assessed in several studies [16, 19, 21–23], very few studies have evaluated the impact of neostigmine on GRV in ICU patients [18, 24, 25]. Moreover, complications such as dysrhythmia and extrapyramidal side effects limit the use of these drugs [18]. Given the above information and the high potential outcome of GRV on mortality in ICU patients and few studies to compare the efficacy of neostigmine and metoclopramide in improving the gastrointestinal feeding intolerance in critically ill patients, this study was aimed at comparing the effects of neostigmine and metoclopramide on GRV in mechanically ventilated ICU patients.

## 2. Methods

### 2.1. Trial Setting

This double-blind randomized clinical trial study was carried out in the Khatam-al-Anbya Hospital in Zahedan, Iran, from August 2019 to September 2020 to compare the effects of neostigmine and metoclopramide on GRV in mechanically ventilated ICU patients. This study was approved by the research ethics committee of the Zahedan University of Medical Sciences, Zahedan, Iran (IR.ZAUMS.REC.1398.185). This trial has also been registered in the Iranian Registry of Clinical Trials (IRCT20190804044432N1). In addition, informed written consent has been obtained from each patient or their legal guardian. All parts of the study were reviewed according to the Consolidated Standards of Reporting Trials (CONSORT) statement [26].

### 2.2. Participants and Eligible Criteria

A total of 200 mechanically ventilated ICU patients of both sexes, aged 20-50 years, with nasogastric tube feeding and GRV > 120 ml (60% of the gavage volume in the previous 6 hours) was enrolled in this prospective double-blind randomized clinical trial. Patients with a history of diabetes, heart block, bradycardia (heart rate < 60/min), using beta-blockers, systolic blood pressure less than 90 mmHg, hypothermia (core temperature below 35° C), renal failure (serum creatinine level > 1.5), using any prokinetic agents such as erythromycin or cisapride within 8 hours prior to beginning of study, recent surgery on the stomach or digestive system within the last ten days, signs and symptoms of intestinal obstruction, pregnancy and lactation, active bronchospasm, occurrence of extrapyramidal side effects, known sensitivity to neostigmine or metoclopramide, and active gastrointestinal (GI) bleeding were excluded from the study.

### 2.3. Sample Size

The sample size of this study was calculated based on the simple formula for the difference in proportions, according to similar studies (*P*_1_ = 0.55and*P*_2_ = 0.28);*Z*_1−*β*_represented the desired power typically 0.84 for 80% power, and*Z*_1−*α*/2_represented the desired level of statistical significance typically 1.96. According to the nature of the clinical trial study and the probability of a sample size drop, a 10% drop was considered as the attrition rate and the final sample size for each group was considered to be 50 subjects. However, we were able to have 100 patients in each group.

(1) $$\begin{array}{c} n=\frac{{\left(Z_{1-\alpha /2}+Z_{1-\beta }\right)}^{2}\left[P_{1}\left(1-P_{1}\right)+P_{2}\left(1-P_{2}\right)\right]}{{\left(P_{1}-P_{2}\right)}^{2}}. \end{array}$$

### 2.4. Randomization and Intervention Procedure

Patients who meet the inclusion criteria were selected through convenient sampling and randomly assigned into two groups (A and B) with 100 patients in each group by a nurse who was blind to the study groups. Block randomization was performed using the sealed envelope technique and computer-generated random numbers by Random Allocation Software© (RAS; Informer Technologies, Inc., Madrid, Spain). At baseline, patients in groups A and B received intravenous infusion of neostigmine at a dose of 2.5 mg/100 ml normal saline and metoclopramide at a dose of 10 mg/100 ml normal saline, within 30 minutes, respectively. Patients' GRVs were evaluated before intervention and 3, 6, 9, and 12 hours after the intervention using a gavage syringe by an expert nurse who had been unaware of the groups under study. Enteral feeding intolerance was defined as GRV > 120 ml. Type and rate of enteral feeding nutrition were same for all patients (180 ml/3 h). All patients have 45-degree head up position.

### 2.5. Data Collection

In addition of evaluating the GRV five times for each patient, demographic and clinical data of the participants, age, gender, diabetes mellitus, opioid, Midazolam uses in each group, Acute Physiology and Chronic Health Evaluation (APACHE) II, and Sequential Organ Failure Assessment (SOFA) score were recorded using a written questionnaire at the beginning of the study.

### 2.6. Statistical Analysis

All the collected data were entered in standard format into SPSS (Statistical Package for the Social Sciences) version 21 software, for further analysis. Data were expressed as mean ± standard division (SD) for continuous variables and percentage (%) for categorical characteristics. The Shapiro-Wilk test was used to test whether data were normally distributed. Baseline demographic and clinical characteristics between the groups (neostigmine and metoclopramide) were assessed using *t*-test or Mann-Whitney *U* test for continuous variables and *χ*^2^ or Fisher's exact tests for comparing categorical proportions. Generalized estimating equations (GEE) were performed on the longitudinal data, and the results were expressed as odds ratios (ORs). The GEE model was used to estimate the differences in values of the GRV state (binary variable) at each time point between the two groups and also the time trend after treatment. A *P* value of 0.05 or less was considered as statistically significant.

## 3. Results

### 3.1. Study Participants

**Figure 1:**
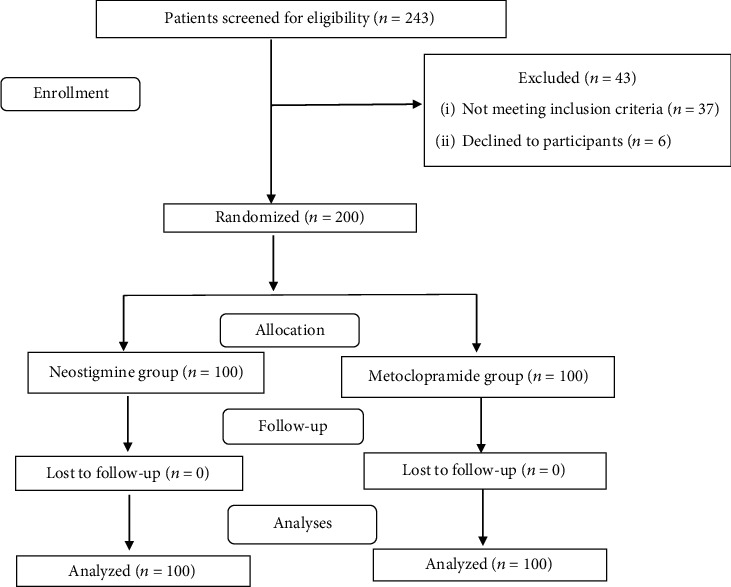
Flow chart of study population selection.

The enrollment flow chart of patients is presented in Figure 1. One hundred cases, in each group, completed the study. Two hundred forty-three mechanically ventilated ICU patients were screened for eligibility criteria. Out of the 243 ICU patients, 200 patients met the inclusion criteria and were randomly assigned into two groups with 100 patients in each group. During the intervention and follow-up stages, no patient was excluded from the study, and finally, 100 patients in each group were analyzed.

### 3.2. Baseline Characteristics of Study Participants

Baseline characteristics of the participants in the two groups of study are presented in Table 1. Baseline characteristics were well matched between the two study groups, and we did not find any significant differences between the two groups of study (*P* > 0.05). The mean ± SD age of the participants were 46.89 ± 1.40 and 46.88 ± 1.46 years in the neostigmine and metoclopramide groups, respectively (*P* = 0.953). In terms of gender, 71 participants (71.0%) in the neostigmine group and 69 (69.0%) in the metoclopramide group were male (*P* = 0.758). In terms of diabetes mellitus, 22 (22%) patients and 23 (23%) patients had diabetes in the neostigmine and metoclopramide groups, respectively. No significant difference was observed between groups (*P* = 0.866). In terms of opioid, 22 (22%) patients and 24 (24%) patients had opioid in the neostigmine and metoclopramide groups, respectively. No significant difference was observed between groups (*P* = 0.737). Additionally, there was no significant difference in the mean score of severity illness based on APACHE II and SOFA between the two groups (15.95 ± 1.78 vs. 15.79 ± 1.87, *P* = 0.537, and 8.17 ± 2.19 vs. 8.31 ± 2.55, *P* = 0.678), respectively. In terms of using Midazolam, we did not find any difference between the two groups (*P* = 0.304).

### 3.3. Adverse Events between Two Groups

Frequency of complications in the two groups of study is presented in Table 2. According to the results, bradycardia was observed in 23% and 21% of patients who received neostigmine and metoclopramide, respectively. In addition, hypertension was reported in 26% and 22% of patients in the neostigmine and metoclopramide groups, respectively. However, these differences were not statistically significant between the two groups in terms of bradycardia (*P* = 0.733) and hypertension (*P* = 0.508). Moreover, VAP occurred only in 2 (2%) and 3 (3%) patients in the neostigmine and metoclopramide groups, respectively. No significant difference was observed between groups (*P* = 0.651).

### 3.4. The Efficacy of Interventions on GRV

**Figure 2:**
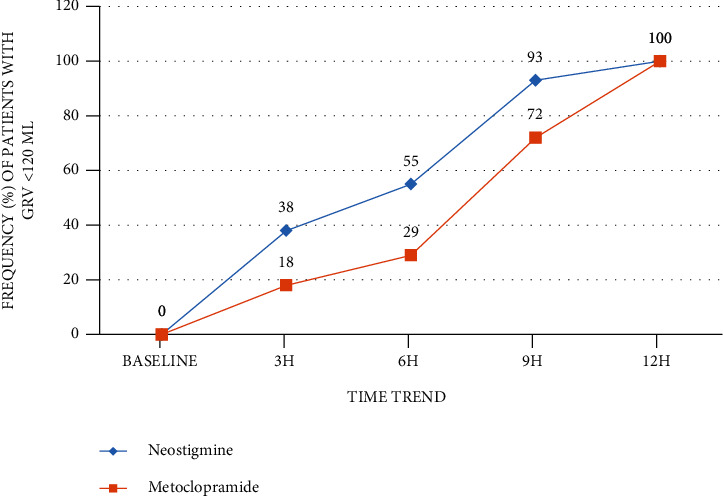
Time trend of GRV improvement in neostigmine and metoclopramide groups.

GRV data was collected preintervention and every three hours for 12 hours of postintervention. GRV improvement status (<120 ml) was evaluated at 3, 6, 9, and 12 hours after the intervention to compare with preintervention (>120 ml); the results for two groups of study are presented in Table 3. After adjusting of demographic and clinical characteristics (age, gender, and SOFA score), the generalized estimating equation (GEE) model revealed that neostigmine treatment increased odds of GRV improvement compared to the metoclopramide group (OR = 2.45, 95% CI: 1.60-3.76, *P* < 0.001). However, there is a statistically significant time trend (within-subject differences or time effect) regardless of treatment groups (*P* < 0.001). The median time required from intervention to recovery of GRV in 50% of patients was 6 hours in the neostigmine group, while for the metoclopramide group, this time was 9 hours, and this difference between the two groups was statistically significant (*P* < 0.001). However, within 12 hours of treatment, all patients in both groups had complete recovery. The time trend of GRV improvement in the neostigmine and metoclopramide groups is shown in Figure 2.

## 4. Discussion

Critically ill, mechanically ventilated patients in the ICU are most likely to experience delayed gastric emptying, intolerance of EN, higher chances of malnutrition [27], pulmonary aspiration, infections [28], and mortality [29, 30]. Therefore, reducing GRV is essential to control these complications. In the present study, we compared the effects of neostigmine and metoclopramide on the GRV of ICU patients. According to the results, although neostigmine treatment significantly increased odds of GRV improvement in more patients in less time, within 12 hours of treatment, all patients in both groups had complete recovery. Moreover, given that the complications were the same in both treatments, it seems that both drugs are effective in improving the GRV for ICU patients. However, the better efficacy of neostigmine in reducing GRV in a shorter period of time compared to metoclopramide is very important for critically ill patients in the ICU, which cannot be ignored.

Neostigmine is a peripheral inhibitor of cholinesterase and has a plasma half-life of 20-60 minutes following intravenous (IV) administration. It produces a smooth contraction of the muscle that triggers an increase in the gut wall's cholinergic activity, which is also believed to promote colonic motility. It was basically used in patients with postoperative ileus, ileus-effect drug intoxication, and colonic pseudoobstruction [16, 19]. In addition, after administration of neostigmine, increased amplitude in electrogastrography was clearly shown [31]. However, in a pilot study by Lucey et al. [18], the effect of neostigmine to increase gastric emptying in critically ill patients was examined, and they suggested that although neostigmine may have a positive effect on gastric emptying and EN absorption in critically ill patients, the results were not statistically significant. Aghadavoudi et al. [24] investigated the direct effect of neostigmine on the tolerance of enteral feeding in patients in the ICU by the evaluation of related factors such as constipation, diarrhea, vomiting, and volume of gastric lavage and obtained results quite similar to the previous study [18] that showed that although the GRV in patients who received neostigmine infusion was lower than that in control group (43.3% versus 63.3%), this difference was not statistically significant.

Metoclopramide is a centrally acting antiemetic, which increases gastric motility via muscarinic receptors [32]. Usually, intravenous metoclopramide is used to monitor delayed gastric emptying and to encourage early enteral feeding [33]. Metoclopramide tachyphylaxis occurs rarely after a couple of days of treatment. The etiology of tachyphylaxis is unclear, but neurohumoral receptor desensitization, down-regulation, and endocytosis have been proposed as mechanisms underlying the occurrence of tachyphylaxis [34]. Similar to our study, a study conducted by Gholipour et al. [25] compared the effect of neostigmine and metoclopramide on GRV in mechanically ventilated ICU patients which showed that neostigmine is more effective than metoclopramide in reducing GRV and improving gastric emptying in ICU patients without significant complication. Our study had a similar outcome. In our investigation, the better efficacy of neostigmine was observed in reducing GRV in a shorter period of time compared to metoclopramide In addition, there was no significant difference between the two groups in terms of complications. Although the incidence of bradycardia and hypertension in patients who received neostigmine infusion was higher than the metoclopramide group, this difference was not statistically significant. Thus, it is important to note that treatment with neostigmine is not without risk. Studies have reported different results on the effect of neostigmine on the length of ICU stay and duration of mechanical ventilation. In a study that was conducted by Aghadavoudi et al. [24], it has been shown that neostigmine reduces the time of hospitalization in the ICU. However, in a study by Gholipour et al. [25], the ICU length of stay and MV duration were slightly higher in the neostigmine group although not statistically significant. In this study, the lack of ICU length of stay and MV duration is a potential limitation of this study. Of course, further evaluation of the effect of neostigmine on the tolerance of enteral feeding is a high potential subject for investigation and practical usage.

The principal strength of this study was that it used several methods to decrease the impact of confounding variables on our research including matching, statistical control, and randomization. However, the limitation of this study is the sample size which was small. Accordingly, a larger sample size study is recommended and further studies to evaluate patients' gastric residual volume using more accurate and precise methods.

## 5. Conclusion

According to the results of the present study, although neostigmine treatment significantly increased the odds of GRV improvement in more patients in less time, within 12 hours of treatment, all patients in both groups had complete recovery. Moreover, given that the complications were the same in both treatments, it seems that both drugs are effective in improving the GRV for ICU patients. However, the better efficacy of neostigmine in reducing GRV in a shorter period of time compared to metoclopramide is very important for critically ill patients in the ICU, which cannot be ignored.

## Figures and Tables

**Table 1:** Baseline characteristics of the participants in two groups of study.

Variables	Groups of study		*P* value
	Neostigmine (*n* = 100)	Metoclopramide (*n* = 100)	
Age, mean ± SD (years)	46.89 ± 1.40	46.88 ± 1.46	0.953
Gender (%)			
Male	71 (71.0)	69 (69.0)	0.758
Female	29 (29.0)	31 (31.0)	
Diabetes mellitus (yes, %)	22 (22.0)	23 (23.0)	0.886
Opioid (yes, %)	22 (22.0)	24 (24.0)	0.737
SOFA, mean ± SD	8.17 ± 2.19	8.31 ± 2.55	0.678
APACHE II, mean ± SD	15.95 ± 1.78	15.79 ± 1.87	0.537
Midazolam (yes, %)	33 (33.0)	40 (40.0)	0.304

**Table 2:** Frequency of complications in two groups of study.

Side effects	Groups of study		Total (*n* = 200)	*P* value
	Neostigmine (*n* = 100)	Metoclopramide (*n* = 100)		
*Bradycardia (%)*				
Yes	23 (23.0)	21 (21.0)	44 (22.0)	0.733
No	77 (77.0)	79 (79.0)	156 (78.0)	
*Hypertension (%)*				
Yes	26 (26.0)	22 (22.0)	48 (24.0)	0.508
No	74 (74.0)	78 (78.0)	152 (76.0)	
*VAP*				
Yes	2 (2.0)	3 (3.0)	5 (2.5)	0.651
No	98 (98.0)	97 (97.0)	195 (97.5)	

**Table 3:** Gastric residual volume (GRV) improvement at 3-, 6-, 9-, and 12-hour follow-up in two groups of study.

GRV	Time trend					*P* value^∗∗^	*P* value^∗∗∗^
	Baseline	3 h	6 h	9 h	12 h		
*Neostigmine*							
<120 cc	0 (0)	38 (38.0)	55 (55.0)	93 (93.0)	100 (100)	<0.001	0.012
>120 cc	100 (100)	62 (62.0)	45 (45.0)	7 (7.0)	0 (0)		
*Metoclopramide*							
<120 cc	0 (0)	18 (18.0)	29 (29.0)	72 (72.0)	100 (100)		
>120 cc	100 (100)	82 (82.0)	71 (71.0)	28 (28.0)	0 (0)		
*P* value^∗^	1	0.003	<0.001	<0.001	1		

^∗^Independent *t*-test between two groups; ^∗∗^repeated measurement of time trend in the GEE model; ^∗∗∗^comparison of change from respective baseline between the neostigmine and metoclopramide groups using the GEE model to control for time effect and sex and gender stay period in the repeated measurement.

## Data Availability

All data generated or analyzed during this study are included in this article. More information concerning the data can be obtained from the corresponding author.
